# A novel adenovirus of Western lowland gorillas (*Gorilla gorilla gorilla*)

**DOI:** 10.1186/1743-422X-7-303

**Published:** 2010-11-05

**Authors:** Diana Wevers, Fabian H Leendertz, Nelly Scuda, Christophe Boesch, Martha M Robbins, Josephine Head, Carsten Ludwig, Joachim Kühn, Bernhard Ehlers

**Affiliations:** 1 FG12 Division of Viral Infections, Robert Koch Institute, Berlin, Germany; 2 Junior research group "Emerging Zoonoses", Robert Koch Institute, Berlin, Germany; 3 Department of Primatology, Max-Planck-Institute for Evolutionary Anthropology, Leipzig, Germany; 4 Westfälischer Zoologischer Garten Münster GmbH, Münster, Germany; 5 Institute of Medical Microbiology, Clinical Virology, University of Münster, Münster, Germany

## Abstract

Adenoviruses (AdV) broadly infect vertebrate hosts including a variety of primates. We identified a novel AdV in the feces of captive gorillas by isolation in cell culture, electron microscopy and PCR. From the supernatants of infected cultures we amplified DNA polymerase (DPOL), preterminal protein (pTP) and hexon gene sequences with generic pan primate AdV PCR assays. The sequences in-between were amplified by long-distance PCRs of 2 - 10 kb length, resulting in a final sequence of 15.6 kb. Phylogenetic analysis placed the novel gorilla AdV into a cluster of primate AdVs belonging to the species Human adenovirus B (HAdV-B). Depending on the analyzed gene, its position within the cluster was variable. To further elucidate its origin, feces samples of wild gorillas were analyzed. AdV hexon sequences were detected which are indicative for three distinct and novel gorilla HAdV-B viruses, among them a virus nearly identical to the novel AdV isolated from captive gorillas. This shows that the discovered virus is a member of a group of HAdV-B viruses that naturally infect gorillas. The mixed phylogenetic clusters of gorilla, chimpanzee, bonobo and human AdVs within the HAdV-B species indicate that host switches may have been a component of the evolution of human and non-human primate HAdV-B viruses.

## Findings

Adenoviruses are non-enveloped icosahedral double-stranded DNA viruses that infect fish, amphibians, reptiles, birds and mammals [1]. Human adenoviruses (HAdV) are categorized into seven species (HAdV-A to HAdV-G) [2]. Each species includes a distinct number of serotypes [3]. In addition, intra-species shuffling of penton base, fiber and hexon genes by recombination has been frequently observed [4-6]. Simian adenoviruses have been discovered in monkeys and great apes [7-11]. They are very similar to HAdV, and most of them can be grouped into corresponding HAdV species or the newly established species Simian adenovirus A (SAdV-A).

In 2008, a group of Western lowland gorillas (*Gorilla gorilla gorilla*) suffered from prolonged diarrhea and self-limiting respiratory disease in the Zoological gardens of Münster, Germany. To isolate viral agents potentially responsible for the symptoms, fecal samples were suspended in phosphate-buffered saline, sterile filtered and cultured on MRC-5 cells and A549 cells. After eight days of culture, a cytopathogenic effect was observed. The culture supernatant was examined by electron microscopy, and virus-like structures were detected their size and general structure being consistent with that of adenoviruses (Additional Figure 1).

**Figure 1:**
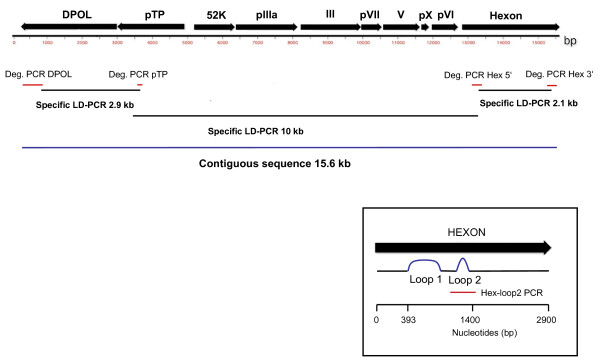
**PCR amplification strategy**. Above the ruler, the amplified part of the AdV genome is depicted. Below, a schematic visualisation of the positions of the degenerated consensus PCRs (red lines), the long-distance PCRs (black lines) and the resulting contiguous sequence (blue line) is given. In the box, the hexon gene is magnified, and the positions of the hexon loops and the Hex-loop2 PCR (red line) are illustrated.

DNA was then extracted from culture supernatant using the Qiagen tissue kit according to the manufacturer's instructions (Qiagen, Hilden, Germany), and a generic primate adenovirus PCR was performed. For this purpose, a nested set of degenerate and deoxyinosine-substituted (deg/dI) primers was designed, targeting a highly conserved region of the DNA polymerase (DPOL) gene of primate mastadenoviruses (Figure 1; Table 1). PCR was performed in a total volume of 25 μl with 0.2 μl AmpliTaq Gold (Applied Biosystems), 20 pmol of each primer, 200 μM dNTPs, 2 mM MgCl_2_, and 5% DMSO. A T-Gradient thermocycler from Biometra was used with the following cycling conditions: 95°C for 12 min, and 45 cycles of 95°C for 30 sec, 45°C (1^st ^round and 2^nd ^round) for 30 sec and 72°C for 2 min, followed by a 15 min final extension step at 72°C. PCR products were purified using the Invisorb DNA clean up kit according to the instructions of the manufacturer (Invitek, Berlin, Germany), and directly sequenced with the Big Dye terminator cycle sequencing kit (Applied Biosystems, Warrington, UK) on a 377 automated DNA sequencer (Applied Biosystems). In BLAST analysis of GenBank, the sequence was most closely related to chimpanzee AdVs and human AdVs of the species HAdV-B. There was less similarity to the six gorilla HAdV-B viruses (SAdV-27.2; SAdV-28.2; SAdV-41.1; SAdV-41.2; SAdV-46; SAdV-47; Table 2) recently described [12]. Since the novel gorilla AdV was the seventh HAdV-B of this host, it was named for the purpose of this paper *Gorilla gorilla *adenovirus B7 (GgorAdV-B7).

**Table 1:** Primers for amplification of DPOL, pTP and hexon gene sequences

Primer-set abbreviation	Targeted gene	Name of primer	sequence 5'-3'	PCR length	**Annealing temp**.
**Degenerate primers**					
DPOL-cons	DPOL	4431-s^**$**^	GTnTwyGAyAThTGyGGhATGTAyGC		
		4428-as	GAGGCTGTCCGTrTC(n/i)CCGTA^#^	956 bp	45°C
		4428-s	CGGACGCCTCTGyTGGAC(n/i)AA		
		4429-as	GGCCAGCACrAA(n/i)GArGC	650 bp	45°C
Hex-5'-cons	Hexon	4515-s	GTGGATGG(n/i)GA(r/i)GG(n/i)TACA		
		4515-as	CGCACAACGTC(r/i)AA(n/i)AC(y/i)TC	536 bp	45°C
		4516-s	TGTAACATGAC(y/i)AA(r(i)GA(y/i)TGG		
		4516-as	CAGGGCCCCCAT(n/i)GACA	381 bp	45°C
Hex3'-cons	Hexon	4517-s	CGCAATGGTC(n/i)TACATGCAC		
		4517-as	CAGTGCCCGA(r/i)TA(k/i)GG(n/i)TT	340 bp	45°C
		4518-s	GCAGGACGC(y/i)TCGGAGTA		
		4518-as	CACCC(k/i)GTT(r/i)TC(n/i)CC	230 bp	45°C
pTP-cons	pTP	4521-s	TGGCGACGT(n/i)GT(n/i)TACAG		
		4521-as	CGGACT(y/i)(k/i)GA(r/i)CCTGAAA	260 bp	45°C
		4522-s	TACAGCCG(n/i)GTSTGGAAC		
		4522-as	CTGAAAGAGAGTTC(n/i)ACAGAATCA	230 bp	45°C
					
**Specific primers for long-distance PCR**					
pTP-DPOL-LD	pTP, DPOL	4659-s	TCGCATCTCCAACGACCT		
		4659-as	GCATCCATGGTGAAGATTCC	2350 bp	60°C
Hex-LD	Hexon	4618-s	AGTTCGCTACACACTGGCTG		
		4618-as	ATTGCGGTGATGATTGAATG	1354 bp	59°C
pTP-Hex-LD	pTP, Hexon	4662-s	CTCGGTATCGTTGACGGC		
		4662-as	GATCAACGGGCACAAAGC	10044 bp	60°C
					
**Primers specific for AdV species B**					
Hex-loop2	Hexon	5442s	GAACAAGATACTTTAGCATGTGGAA		
		5442as	GATTGAATGGATTAACATTGTCC	468 bp	55°C
	Hexon	5443s	TAGAAAATCACGGGGTGGAAGA		
		5443as	GGCATCCAAAGACCATCTG	380 bp	55°C

^$ ^s = sense, as = antisense ^# ^I = inosine

**Table 2:** Adenoviruses, accession numbers and hosts

Adenovirus	Abbreviation	GenBank accession number	Host	Wild (Gabon)	Captive
**HAdV-B of this study**					
Gorilla gorilla adenovirus B7	GgorAdV-B7	HQ292614	Western lowland gorilla	+	+
Gorilla gorilla adenovirus B8	GgorAdV-B8	HQ292615	Western lowland gorilla	+	+
Gorilla gorilla adenovirus B9	GgorAdV-B9	HQ292616	Western lowland gorilla		+
Gorilla gorilla adenovirus B10	GgorAdV-B10	HQ292617	Western lowland gorilla	+	
					
**Published HAdV-B**					
Simian adenovirus 21	SAdV-21	AC000010	Chimpanzee		+
Simian adenovirus 27.1	SAdV-27.1	FJ025909	Chimpanzee		+
Simian adenovirus 27.2	SAdV-27.2	FJ025928	Gorilla		+
Simian adenovirus 28.1	SAdV-28.1	FJ025914	Chimpanzee		+
Simian adenovirus 28.2	SAdV-28.2	FJ025915	Gorilla		+
Simian adenovirus 29	SAdV-29	FJ025916	Chimpanzee		+
Simian adenovirus 32	SAdV-32	FJ025911	Chimpanzee		+
Simian adenovirus 33	SAdV-33	JF025908	Chimpanzee		+
Simian adenovirus 35.1	SAdV-35.1	FJ025912	Chimpanzee		+
Simian adenovirus 35.2	SAdV-35.2	FJ025910	Bonobo		+
Simian adenovirus 41.1	SAdV-41.1	FJ025913	Gorilla		+
Simian adenovirus 41.2	SAdV-41.2	FJ025927	Gorilla		+
Simian adenovirus 46	SAdV-46	FJ025930	Gorilla		+
Simian adenovirus 47	SAdV-47	FJ025929	Gorilla		+
Human adenovirus B3	HAdV-B3	DQ086466	Human		
Human adenovirus B7	HAdV-B7	AC000018	Human		
Human adenovirus B11	HAdV-B11	AY163756	Human		
Human adenovirus B14	HAdV-B14	AY803294	Human		
Human adenovirus B16	HAdV-B16	AY601636	Human		
Human adenovirus B21	HAdV-B21	AY601633	Human		
Human adenovirus B34	HAdV-B34	AY737797	Human		
Human adenovirus B35	HAdV-B35	AY271307	Human		
Human adenovirus B50	HAdV-B50	AY737798	Human		

To acquire extended sequence information of GgorAdV-B7, three additional nested PCR assays were designed (Table 1) targeting the preterminal protein (pTP) and two conserved regions at the 5'- and 3'-end of the hexon gene (Figure 1). PCRs were performed as described above, except that elongation at 72°C was for 1 min. With each primer set products of the expected size were obtained. BLAST analysis of their sequences also revealed a HAdV-B-like virus (not shown). To prove that the DPOL, pTP and hexon sequences originate from the same virus, we connected them with long-distance (LD) PCRs (Figure 1) using the TaKaRa-EX PCR system according to the instructions of the manufacturer (Takara Bio Inc., Otsu, Japan). The LD primer pairs are listed with their annealing temperatures in Table 1. Three overlapping PCR products were generated and sequenced by primer walking. A final contiguous sequence of 15637 bp was obtained spanning the genes DPOL, pTP and 52 k, the genes encoding the AdV proteins pIIIa, III (penton base), pVII, V, pX and pVI, and the hexon gene of GgorAdV-B7 (Figure 1).

**Figure 2:**
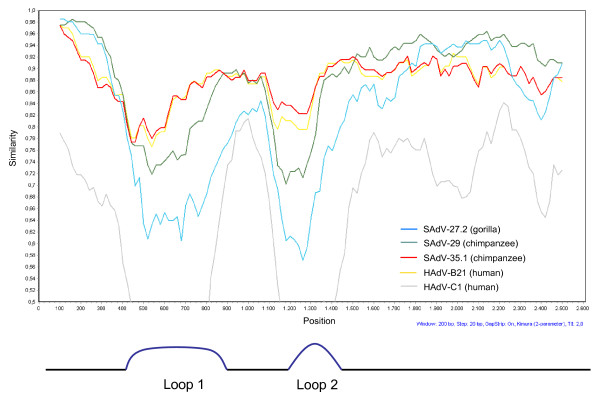
**Simplot analysis of the hexon gene**. 2.8 kb of the GgorAdV-B7 hexon gene were compared to the hexon genes of selected chimpanzee (green and red line), gorilla (blue line) and human (yellow and grey line) AdVs. Below the plot, the analysis parameters are listed in blue font. The hexon Loop 1 and Loop 2 regions are indicated at the bottom.

**Figure 3:**
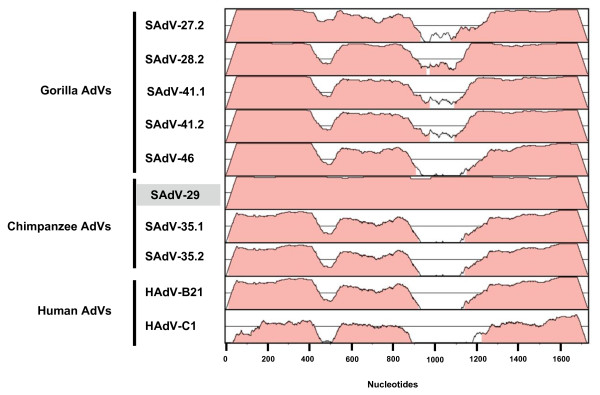
**Pairwise sequence alignment of the penton base open reading frame**. The penton base gene of GgorAdV-B7 was compared with those of five gorilla-, three chimpanzee- and two human AdVs. The 50-100 percent sequence conservation is represented by the height of each data point along the y axis. The chimpanzee AdV SAdV-29 is highlighted in grey designating the exceptionally high similarity to GgorAdV-B7.

Since the AdV hexon gene is an important member of the core gene set used for AdV classification [2,3,13,14], we compared the available partial hexon gene of GgorAdV-B7 (2.7 kb) pair-wise with the corresponding hexon sequences of the most closely related human and chimpanzee HAdV-B viruses and all published gorilla HAdV-B viruses. The highest identity percentages were 96.6% for SAdV-35.1 (chimpanzee AdV) and 96% for HAdV-B21. The gorilla AdV SAdV-27.2, SAdV-28.2, SAdV-41.1, SAdV-41.2, SAdV-46 and SAdV-47 revealed only 87-91% identity. This closer relationship to HAdV-B21 and SAdV-35.1 was restricted to the loop-encoding regions 1 and 2 [15] as visible in an analysis with the software SIMPLOT http://sray.med.som.jhmi.edu (Figure 2). In pair-wise comparisons of DPOL and pTP genes, GgorAdV-B7 was equally closely related to chimpanzee and gorilla HAdV-B viruses (96-99.9%). However, the penton base gene of GgorAdV-B7 showed a striking similarity (99.7%) only to that of SAdV-29 (chimpanzee AdV). Using the program mVISTA http://genome.lbl.gov/vista/index.shtml, the near-perfect match of the GgorAdV-B7 and SAdV-29 penton base genes over the entire gene length is clearly visible (Figure 3).

**Figure 4:**
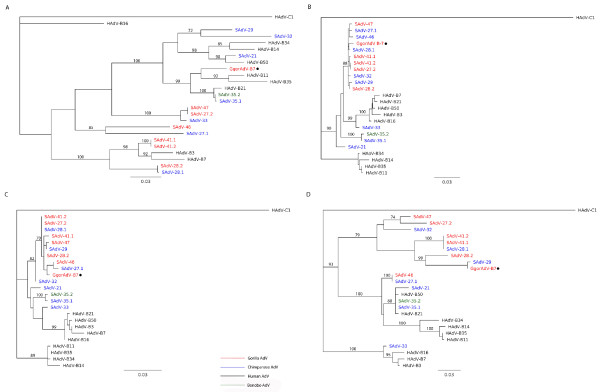
**Phylogenetic analysis**. The Hexon (A), DPOL (B), pTP (C) and penton (D) sequences of GgorAdV-B7 and those of published NHP and human HAdV-B viruses were aligned and subjected to phylogenetic analysis. Human, chimpanzee, bonobo and gorilla AdVs are in black, blue, green and red font, respectively. AdVs discovered in this study are marked with black dots.

With the PhyML plug-in 2.0.1 of the Geneious Pro 5.0.4 software, phylogenetic trees were constructed on the basis of hexon, DPOL, pTP and penton base gene alignments (Figures 4). All published, completely sequenced HAdV-B viruses were included. In the hexon-based tree, GgorAdV-B7 clustered with HAdV-B21, SAdV-35.1 (chimpanzee AdV) and SAdV-35.2 (bonobo AdV) (Figure 4a). DPOL and pTP analyses placed GgorAdV-B7 into a tight cluster of gorilla and chimpanzee AdVs (Figure 4b and 4c). In the tree derived from the penton base, GgorAdV-B7 branched separately, nearly at the same position as SAdV-29 (Figure 4d). With the MrBayes 2.0.2 plug-in (Geneious Pro) or the Neighbor-Joining module of MacVector 10.6, trees with the same AdV clusters and similarly supported topology were obtained (data not shown).

The remarkably close relatedness of GgorAdV-B7 to chimpanzee AdVs (SAdV-29 and 35.1) prompted us to investigate whether gorillas naturally host GgorAdV-B7. For this purpose, we examined wild Western lowland gorillas (*Gorilla. g. gorilla*) from Gabon and additional captive gorillas. Fecal samples were collected from 19 individuals in a remote area with little human presence in Loango National Park, Gabon. They originated from fresh nest sites or were freshly found on gorilla paths [16]. Samples were collected using single-use gloves and preserved by drying over silica. DNA was extracted following a previously described method [17]. In addition, ten necropsy samples (spleen, liver, pancreas, lymph node, tonsil, lung, kidney, urine) and one plasma sample were collected from four deceased captive gorillas in the Zoological gardens of Berlin as well as six fecal samples from three captive gorillas in the Zoological gardens of Münster, Germany. To test for the presence of HAdV-B viruses, we set up a nested PCR (PCR Hex-loop2; Table 1) which targets flanking sequences of a hyper variable region (loop 2) in the hexon gene (Figure 1) and amplifies 380 bp. The primers were deduced from HAdV-B sequences only and not degenerated. A total of 36 gorilla samples were screened. AdV DNA was only detected in feces (5/19 wild gorilla samples and 4/6 captive gorilla samples). In total, 9/25 fecal samples were PCR-positive (36%), and the products sequenced. Most importantly, a virus apparently identical to GgorAdV-B7 was identified in a wild gorilla from Gabon. Three additional HAdV-B viruses were also detected. Two were without close similarities to any published AdV sequence. The third one was nearly identical to the gorilla AdVs SAdV-27.2 and SAdV-47, which had been originally isolated from captive individuals [12]. They were tentatively named GgorAdV-B8, -B9 and -B10.

**Figure 5:**
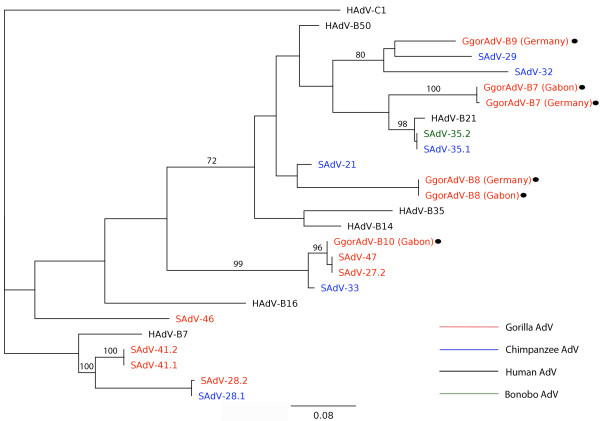
**Phylogenetic analysis of the hexon loop 2 region**. For details, see legend of Figure 4.

GgorAdV-B8 was detected in two fecal samples from wild gorillas in Gabon and in one sample from Münster. Its hexon sequence revealed the highest percentage of identity (86.5%) to SAdV-21 (chimpanzee HAdV-B). GgorAdV-B9 was only amplified from captive gorillas (two fecal samples from Münster) and showed 88% identity to SAdV-29 (chimpanzee HAdV-B). The GgorAdV-B7 to -B10 Hex-loop2 sequences and closely related sequences of published gorilla, chimpanzee, bonobo and human HAdV-B viruses were subjected to phylogenetic analysis as described above. In the tree, the HAdV-B viruses segregate into several subclades with members of two, three or four host species (human, chimpanzee, bonobo and gorilla) (Figure 5). This mixed clustering was also observed upon analysis of the nearly complete hexon gene (2.7 kb; Figure 4a) with GgorAdV-B7 only. It was partially visible in the penton base tree (Figure 4d) and entirely absent from the DPOL and pTP trees (Figures 4b and 4c). In addition, the phylogenetic position of a given primate AdV frequently differed, depending on the analyzed gene (compare Figures 4a to 4d).

Taken together, these observations cannot be explained by co-speciation. Rather, they are in line with recombination and host switching. Such events have been previously discussed to be involved in the evolution of human AdV, because intra-species shuffling of penton base, fiber and hexon genes has been frequently observed [4-6]. In addition, a recombinant between viruses of the sub-clades HAdV-B1 and HAdV-B2 has been isolated from a captive chimpanzee [12]. Here, a close similarity of GgorAdV-B7 to SAdV-29 (complete sequence, excluding hexon loops) and to SAdV-35.1 (hexon loops) was observed (Figure 2). However, since in the loop region the nucleic acid identity between GgorAdV-B7 and SAdV-35.1 was well below 100%, it is unlikely that the existing AdVs are parent viruses in a recent recombination event giving rise to GgorAdV-B7. Rather, a more ancient one with subsequent genetic drift may have been involved or recombination with an unknown AdV, as suggested for HAdV-A18 [18].

Shuffling of genes by recombination between AdVs that naturally infect different host species (e.g., great ape and human AdVs) but under certain conditions co-infect the same host, may be an additional mechanism by which AdVs exchange genetic information. This could occur in places where contacts between humans and apes are frequent like in zoos and animal facilities. In addition, people who are involved in hunting primates and preparation of bush meat [19] are at risk to be infected. So far, infections of humans with non-human primate (NHP) AdVs have not been observed. Nevertheless, antibodies with specificity for chimpanzee HAdV-C viruses have been detected in humans from Sub-Saharan Africa and were significantly less frequent in people from the United States of America and Thailand [20]. In addition, the species HAdV-E comprises only one human serotype but more than 12 great ape serotypes. Therefore, the human HAdV-E was thought to be the result of a zoonotic transmission from chimpanzees to humans [21]. The gorilla AdV described in the present study (GgorAdV-B7) is highly similar to chimpanzee AdVs. Thus, a transmission event between chimpanzees and gorillas was possibly involved which is further indication for the potential of AdVs to jump between closely related hosts.

Very little is known about the pathogenic properties of NHP-AdV. GgorAdV-B7 was originally discovered in a group of gorillas suffering from prolonged diarrhea and self-limiting respiratory infection. Since human species B AdV have been linked to respiratory diseases [22-24], an etiological association of GgorAdV-B7 with the observed respiratory symptoms is possible. However, recent studies reported the frequent shedding of AdVs in the feces of healthy captive chimpanzees and gorillas [12,25]. Therefore, further investigations are needed.

Knowledge about the spectrum of AdV in wild great apes in general [25] is very limited. Specifically from wild gorillas, no information has been published. Although examining only a small set of samples, our findings show that AdV infecting captive gorillas can readily be found in wild animals (GgorAdV-B7; GgorAdV-B8). This is a good example of how humans may be brought into contact with new pathogens, not only locally through bushmeat hunting in regions where NHP live naturally, but also in other regions of the world where NHP are housed in zoos.

The high variety of known and novel HAdV-B viruses in great apes calls for larger studies to understand the diversity of AdVs currently circulating in African NHP as well as in local human populations. It is justified to assume that such studies will improve our insight into the zoonotic potential of adenoviruses and possibly answer the intriguing question whether AdVs of non-human primates have already contributed to the human "adeno-virosphere".

## Accession numbers

The sequences reported in this study were deposited in GenBank under the accession numbers listed in Table 2.

## Competing interests

The authors declare that they have no competing interests.

## Authors' contributions

DW and JK performed the cell culture experiments. DW, NS, JK and JH performed the molecular genetic studies. FHL, CB and MMR coordinated field work and sample collection. JH and CL sampled gorilla feces. DW, NS, JK, FHL and BE conceived of the study, and participated in its design and coordination. DW, FHL, CB, JK and BE drafted the manuscript. All authors read and approved the final manuscript.

## Supplementary Material

Additional Figure 1 **Negative stain electron micrograph of adenovirus-like particles isolated from a fecal sample of a captive gorilla**. Negatively stained with 1% uranyl acetate. Virus particles are 70-90 nm in diameter with an icosahedral shape. Scale bar = 200 nm. Click here for file
