# Supplementary figures and images for: A novel adenovirus of Western lowland gorillas (Gorilla gorilla gorilla)
Source: Virol J. 2010 Nov 5;7:303. doi: 10.1186/1743-422X-7-303 (PMC2989969; doi:10.1186/1743-422X-7-303)

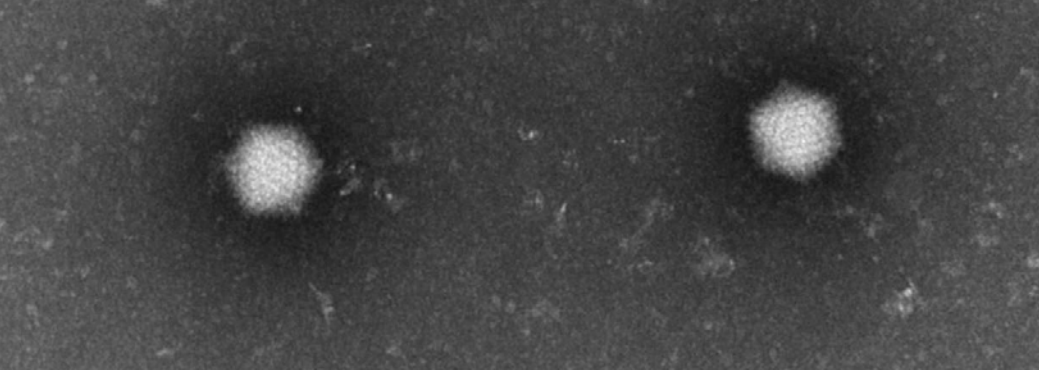

Supplement: Additional Figure 1 — Negative stain electron micrograph of adenovirus-like particles isolated from a fecal sample of a captive gorilla. Negatively stained with 1% uranyl acetate. Virus particles are 70-90 nm in diameter with an icosahedral shape. Scale bar = 200 nm. [file 1743-422X-7-303-S1.PDF]
